# Involvement of collagen-binding heat shock protein 47 in scleroderma-associated fibrosis

**DOI:** 10.1007/s13238-015-0171-3

**Published:** 2015-06-20

**Authors:** Haiyan Chu, Ting Wu, Wenyu Wu, Wenzhen Tu, Shuai Jiang, Sidi Chen, Yanyun Ma, Qingmei Liu, Xiaodong Zhou, Li Jin, Jiucun Wang

**Affiliations:** Ministry of Education Key Laboratory of Contemporary Anthropology and State Key Laboratory of Genetic Engineering, Collaborative Innovation Center for Genetics and Development, School of Life Sciences and Institutes of Biomedical Sciences, Fudan University, Shanghai, 200438 China; Division of Dermatology, Huashan Hospital, Fudan University, Shanghai, 200040 China; Division of Rheumatology, Shanghai TCM-Integrated Hospital, Shanghai, 200082 China; Division of Rheumatology, University of Texas Health Science Center at Houston, Houston, TX 77030 USA; Institute of Rheumatology, Immunology and Allergy, Fudan University, Shanghai, 20080 China

**Keywords:** systemic sclerosis, fibrosis, collagen, heat shock protein 47, anti-centromere antibody, therapeutic target

## Abstract

Uncontrolled fibrosis of skin and internal organs is the main characteristic of scleroderma, and collagen is a major extracellular matrix protein that deposits in the fibrotic organs. As the chaperone of collagen, heat shock protein 47 (HSP47) is closely related with the development of fibrosis. To explore the potential function of HSP47 in the pathogenesis of scleroderma, the clinical, *in vivo* and *in vitro* studies were performed. In clinical, the increased mRNA level of HSP47 was observed in the skin fibroblasts and PBMC from scleroderma patients, and the enhanced protein level of HSP47 was also detected in the skin biopsy and plasma of the above patients. Unexpectedly, the enhanced levels of HSP47 were positively correlated with the presence of anti-centromere antibody in scleroderma patients. Moreover, a high expression of HSP47 was found in the skin lesion of BLM-induced scleroderma mouse model. Further *in vitro* studies demonstrated that HSP47 knockdown could block the intracellular and extracellular collagen over-productions induced by exogenous TGF-β. Therefore, the results in this study provide direct evidence that HSP47 is involved in the pathogenesis of scleroderma. The high expression of HSP47 can be detected in the circulatory system of scleroderma patients, indicating that HSP47 may become a pathological marker to assess the progression of scleroderma, and also explain the systemic fibrosis of scleroderma. Meanwhile, collagen over-expression is blocked by HSP47 knockdown, suggesting the possibility that HSP47 can be a potential therapeutic target for scleroderma.

## INTRODUCTION

Systemic sclerosis (scleroderma, SSc) is a complex autoimmune disease in which excessive accumulation of extracellular matrix (ECM) in the skin and internal organs is a major pathological finding. Although the etiology of this disease remains unknown, a number of therapeutic approaches are available for treating scleroderma, including circulation improvement, blocking production of certain pro-inflammatory and pro-fibrotic cytokines, as well as fibrosis prevention and treatment (Bournia et al., 2009; Sapadin and Fleischmajer, 2002), due to vascular, immunologic and fibrotic alterations in SSc (Denton et al., 2006). However, none of these treatments are consistently effective in attenuating fibrosis (Leask, 2012; Martin et al., 2012; Zandman-Goddard et al., 2005). Therefore, a better assessment marker of the fibrosis activity and comprehensive understanding of the underlying mechanisms of collagen over-production may provide new strategies for the anti-fibrotic treatment of SSc.

HSP47, a 47-kDa heat-shock protein, is initially characterized as a cell surface-associated protein that binds type IV collagen or gelatin, and later identified as a major collagen binding heat-inducible protein in chick embryo fibroblasts (Kurkinen et al., 1984). HSP47 is a collagen-specific molecular chaperone localized in the endoplasmic reticulum (Saga et al., 1987), and belongs to the serpin family, a superfamily of plasma serine protease inhibitors, but lacks the active site essential for the inhibition of proteases (Hirayoshi et al., 1991). During the maturation of collagen protein, HSP47 transiently binds to nascent pro-collagen once it is co-translocated into the endoplasmic reticulum (ER) and facilitates the assembly/secretion of collagens. After the triple helix collagen formation, HSP47 and the triple helix proceed to the Golgi, where HSP47 dissociates from the pro-collagen in the *cis*-Golgi complex (Nagata, 1998).

HSP47 has been reported to be involved in various fibrotic processes (Kakugawa et al., 2010; Masuda et al., 1994), such as the experimental model of liver cirrhosis (Masuda et al., 1994), pulmonary fibrosis (Hagiwara et al., 2007; Kakugawa et al., 2004; Kakugawa et al., 2010; Razzaque et al., 1998), and peritoneal fibrosis (Nishino et al., 2003). As to the association of HSP47 with scleroderma, HSP47 was found highly expressed in cultured fibroblasts from Japanese SSc together with type I collagen, which was correlated with shorter disease duration (Kuroda et al., 1998).

Since HSP47 is pivotal for the production of collagen, which closely related to fibrosis development of SSc, our aim was to explore the role of HSP47 in the regulation of collagen protein expression during fibrosis development of SSc and whether HSP47 would be a new serum marker to assess the fibrosis activity of the disease.

In this report, we provide evidences supporting that HSP47 is indeed implicated in the systemic fibrosis of SSc. The expression of HSP47 is rather higher in the plasma, peripheral blood mononuclear cells (PBMC) and skin biopsy from SSc patients, as well as skin of bleomycin-induced dermal fibrosis model. In addition, the elevated expression of HSP47 is positively correlated with presence of ACA in SSc patients. And the blockade of HSP47 can inhibit the over-production of collagen. Thus, the present data raising the likelihood that HSP47 may be an indicator for fibrosis activity of SSc and presented as a potential therapeutic target for SSc.

## RESULTS

### The expression of HSP47 was increased in the peripheral blood mononuclear cells and plasma from SSc patients

**Figure 1:**
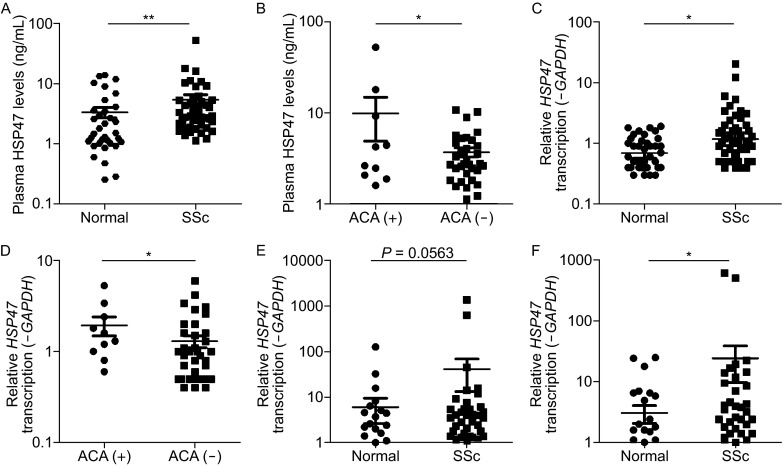
**The protein level of HSP47 in the plasma and the mRNA level of** ***HSP47*** **in the peripheral blood mononuclear cells were both elevated in the SSc**. (A) As assessed by ELISA, the level of HSP47 was elevated in the plasma of SSc patients compared with that of normal people (5.4 ± 0.2 ng/mL vs. 3.3 ± 0.1 ng/mL). (B) The level of HSP47 was elevated in the plasma of patients with anti-centromere antibody compared to patients without anti-centromere antibody (9.9 ± 1.6 ng/mL vs 3.7 ± 0.07 ng/mL). (C) Relative mRNA levels of *HSP47* in the PBMC for SSc (2.0 ± 0.1) vs. normal control (0.8 ± 0.01) using RT-PCR. (D) The relative levels of mRNA of *HSP47* in PBMC of patients with anti-centromere antibody compared to patients without anti-centromere antibody (1.9 ± 0.14 vs. 1.3 ± 0.04). (E) The relative mRNA levels of *COL1A2* in the PBMC were SSc (24.1 ± 2.0) vs. normal (3.1 ± 0.2). (F) The relative mRNA level of *COL3A1* in the PBMC were SSc (41.4 ± 3.8) vs. normal (6.0 ± 0.5). The mRNA levels of those genes were calculated using a relative ratio to *GAPDH*. The y axis is plotted on a logarithmic scale. Values show the means ± SEM. *P < 0.05, **P < 0.01

The peripheral blood mononuclear cells (PBMC) and plasma from SSc patients were also collected to further clarify the function of HSP47 in the development of SSc. The results from ELISA assays showed that the secreted HSP47 was elevated significantly in the plasma of SSc patients compared with that of normal controls (5.4 ± 0.2 ng/mL vs. 3.3 ± 0.1 ng/mL, *P* = 0.0025; Fig. 1A). Furthermore, the HSP47 level in the plasma from the patients with anti-centromere antibody was significantly higher than those without anti-centromere antibody (9.9 ± 1.6 ng/mL vs. 3.7 ± 0.07 ng/mL, *P* = 0.0315; Fig. 1B). The *HSP47* expression was also examined in the PBMC of the above SSc patients, and it was found significantly increased by more than 2-fold (2.0 ± 0.1 vs. 0.8 ± 0.01, *P* = 0.0312; Fig. 1C). Consistently, the mRNA level of *HSP47* was significantly up-regulated in the patients with anti-centromere antibody compared to those without anti-centromere antibody by 1.5-fold (1.9 ± 0.14 vs.1.3 ± 0.04, *P* = 0.0393; Fig. 1D). In addition, the transcription levels of *COL1A2* and *COL3A1* in SSc patients were also markedly up-regulated compared with those in normal controls about 8-fold and 7-fold, respectively (24.1 ± 2.0 vs. 3.1 ± 0.2 and 41.4 ± 3.8 vs. 6.0 ± 0.5, *P* = 0.0365 and *P* = 0.063, respectively; Fig. 1E and 1F). Thus, it can be reasonably assumed that HSP47 is implicated in the pathogenesis of scleroderma.

### HSP47 was up-regulated in the skin lesion samples and skin fibroblasts obtained from SSc patients

**Figure 2:**
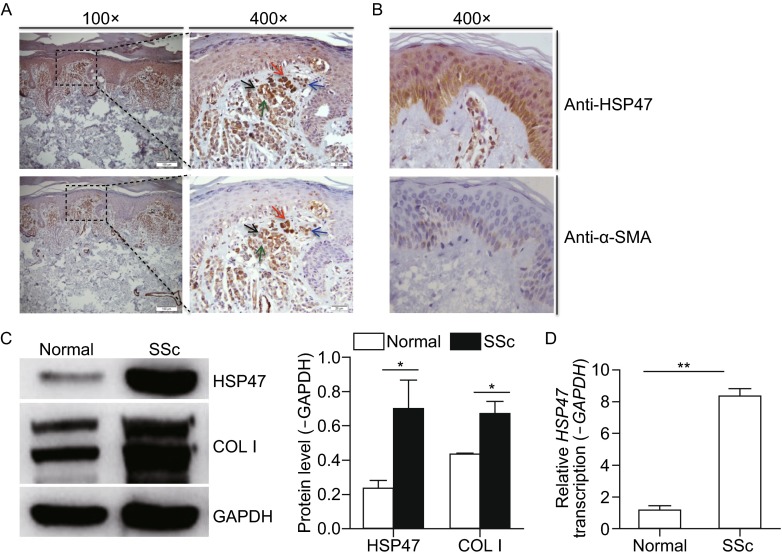
**Expression of HSP47 in normal people and SSc patients’ skin**. (A) IHC for HSP47 and α-SMA in skin biopsy from five SSc patients and HSP47 positive cells were localized on the α-SMA positive cells. (B) IHC for HSP47 and α-SMA in skin biopsy from three normal human. Compared with normal controls, significant increase of HSP47 and α-SMA expression in the skin of SSc patients was observed. Arrows indicate the positive cells, and arrows with the same color indicate the same cells. The length of bars in the figures with an original magnification of 100× are 100 μm, and the length of bars in the figures with an original magnifications of 400× are 20 μm. (C) Western blot analysis revealed increased expression of HSP47 and COL1 in the fibroblasts from the skin of SSc patients, and densitometric analysis results of Western blot for HSP47 and COL I were shown in the column chart. (D) RT-PCR revealed significant increases in the mRNA levels of *HSP47* in the fibroblasts cultured from SSc patients’ skin compared with those from normal controls. *P < 0.05, **P < 0.01 vs. normal control. Experiments were repeated three times

Adjacent 4-µm-thick sections of fibrotic skin from SSc patients were collected for immunohistochemical staining for HSP47 and α-smooth muscle actin (α-SMA). A greater number of positive cells for both HSP47 and α-SMA with a similar expression pattern were observed in SSc patients compared with normal tissues (Fig. 2A and 2B). Meanwhile, HSP47 positive cells were almost the same cells as α-SMA positive staining, and most of those cells were myofibroblasts, indicating that HSP47 was mainly expressed in the cells responsible for collagen synthesis (Fig. 2A). Therefore, high expression of HSP47 was associated with the excessive collagen production in SSc patients.

Fibroblasts were cultured from the skin tissues of Chinese Han SSc patients to measure HSP47 expression. As illustrated in Fig. 2, the protein levels of HSP47 and COL1 were significantly upregulated in the SSc dermal fibroblasts compared with those from normal controls by about 3.5-folds and 1.5-folds, respectively (Fig. 2C). The transcription level of HSP47 was increased by 8-fold and (Fig. 2D). And the expression of TGF-β was also found up-regulated in the cultured skin fibroblast of SSc patients as examined previously (Wu et al., 2014).

### Expression of HSP47 was increased in the scleroderma mouse model

**Figure 3:**
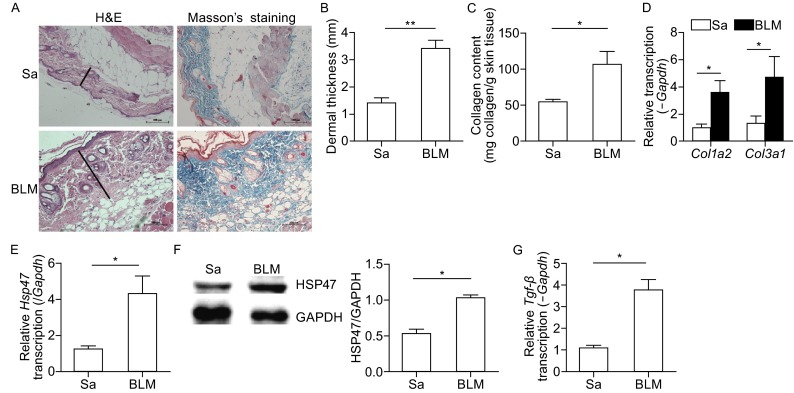
**Expression of HSP47 increased in the skin lesions of mice treated with BLM**. (A) Hematoxylin and eosin (H&E) and Trichromestaining for the skin tissues of normal control mice and BLM-treated mice, Original magnifications were 200×, and the length of bars in the figures are 100 μm; (B) Dermal thickness was calculated at 10× microscopic magnification by measuring the distance between the dermal-epidermal junction and the derma-subcutaneous fat junction (μm) (as indicated by H&E staining of in Fig. 1A) in five randomly selected fields for each skin section. (C) Deposition of collagen in the skin was examined by Sircol assay. RT-PCR was used to analyze the expression of collagen (D), *Hsp47* (E) and *Tgf-β* (G) in the skin of mice, and the mRNA levels were calculated using a relative ratio to *Gapdh*. (F) Western blots for the protein level of HSP47 in mice skin, and densitometric data of Western blot for HSP47 was shown in the column chart. Bars show the means ± SEM results from 6 mice in each group. *P < 0.05, **P < 0.01 versus saline treated group (Sa). Each group *n* = 6

To further demonstrate the role of HSP47 in the pathogenesis of SSc, the scleroderma mouse model was stablished in our study. As demonstrated by our previous study, the histological and molecular hallmarks that are similar to SSc patients are present in the bleomycin-treated mice established in our study (Wang et al., 2010; Wang et al., 2011). As shown in Fig. 1, compared with saline treated mice, BLM induced marked hypodermal thickness (Fig. 3A and 3B), increase of ECM deposition and collagen contents (Fig. 3C and 3D), enhancement of the transcription levels of *Col1a2* and *Col3a1*. Furthermore, HSP47 expression level was examined in this mouse model. There was a significant (5-fold) increase in transcription level of *Hsp47* (Fig. 3E), and a significant (2-fold) increase in the protein level of HSP47 (Fig. 3F). Therefore, HSP47 was highly expressed in the skin tissues from BLM-induced SSc mouse model. Meanwhile, mRNA level of *Tgf-β* was increased in the skin lesion of the SSc mice model (Fig. 3G).

### Inhibition of HSP47 expression may block exogenous TGF-β-induced increases in collagen

**Figure 4:**
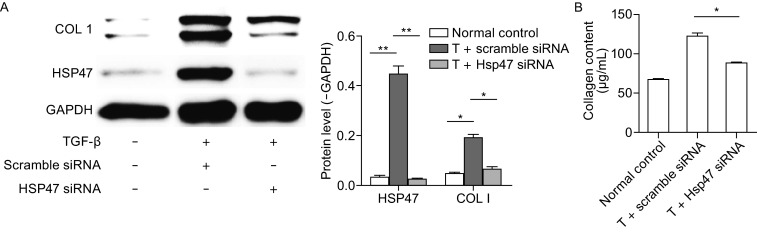
**The effect of HSP47 on the production of collagen**. (A) NIH/3T3 cell was incubated with exogenous TGF-β for 24 h, then transiently transfected with *Hsp47* siRNA for another 24 h, scramble siRNA as control, HSP47 and type I collagen protein were determined by Western blot and the densitometry data of Western blot for HSP47 and type I collagen proteins were shown in the column chart. (B) The collagen content in the cell supernatant was measured by Sircol assay. Values are expressed as means ± SEM. *P < 0.05, **P < 0.01 vs. normal control. Experiments were repeated three times

To further explore the potential role of HSP47 in collagen production during the fibrogenesis of SSc, HSP47 expression was inhibited by corresponding siRNA transfection, and then the intracellular and extracellular collagens were examined by Western blot and Sircol assay, respectively. As shown in Fig. 4A, TGF-β stimulation could increase the protein levels of HSP47 and type I collagen, whereas the collagen expression was decreased by knockdown of *Hsp47* (Fig. 4A). In addition, collagen secretion was also reduced after knockdown of *Hsp47* (Fig. 4B). Therefore, these results strongly suggested that HSP47 could play an essential role in promoting collagen synthesis and secretion during the fibrogenesis of SSc.

### The expression of HSP47 was prominently increased by exogenous TGF-β induction in a dose-dependent manner

**Figure 5:**
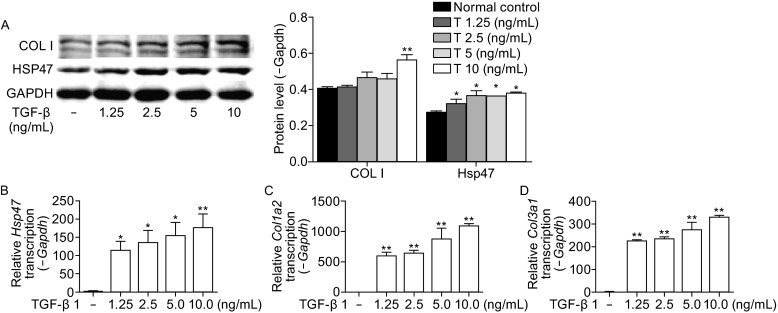
**TGF- β increases expression of HSP47 and collagen in a dose-dependent manner**. (A) Treatment with TGF-β for 48 h increased the protein level of HSP47 and type I collagen in a dose-dependent manner and densitometric analysis of Western blot for HSP47, COLI were shown in the column chart. RT-PCR revealed increased *Hsp47* (B), type I collagen (C), and type III collagen (D) mRNA after treatment with TGF-β for 12 h. Relative mRNA was calculated using a relative ratio to *Gapdh*. Values are expressed as means ± SEM. *P < 0.05, **P < 0.01 vs. normal control. Experiments were repeated three times

TGF-β is a well-known potential pro-fibrogenic cytokine. The NIH/3T3 fibroblasts stimulated by exogenous TGF-β could display SSc-like phenotype (Wu et al., 2014). Given these two reasons, NIH/3T3 fibroblasts were used to test the hypothesis that TGF-β was responsible for the upregulation of HSP47 expression. The results suggested that both HSP47 expression and collagen production were increased by TGF-β induction in a dose-dependent manner, and the maximum level of both the HSP47 expression and collagen production were observed at 10 ng/mL of TGF-β. In addition, the mRNA level of *Hsp47* was enhanced by TGF-β at 12 h and the protein level was elevated at 48 h, respectively (Fig. 5A–D). The results confirmed that the expression of HSP47 could be regulated by TGF-β.

## DISCUSSION

SSc is a complex immune connective tissue disorder with the main characteristic of fibrosis in skin and multiple visceral organs. Despite the fact that numerous hypotheses involving fibrosis development have been investigated, the underlying mechanism has not yet been discovered (Bhattacharyya et al., 2012; Broen et al., 2012; Hunzelmann and Brinckmann, 2010; Martin et al., 2012; Rabquer and Koch, 2012). The uncontrolled synthesis and excessive deposition of collagen are the main characteristics of fibrotic disorders. Thus, a thorough understanding of the mechanism regulating collagen synthesis is essential to develop better therapies to ameliorate fibrosis. HSP47 is a collagen-specific molecular chaperone that is essential for the biosynthesis of collagen molecule (Nagata, 1998), and both *in vivo* and *in vitro* studies have highlighted the importance of HSP47 in the pathogenesis of various fibrotic diseases. The present work was designed to determine the role of HSP47 in fibrogenesis of SSc.

In a previous study reported by Fujimoto M and his colleagues that the HSP47 antibody was elevated in the serum of SSc patients (Fujimoto et al., 2004), however, the expression level of HSP47 protein in the serum was unclear, and our study found that an elevated HSP47 protein level was observed in the plasma of SSc patients, at the same time, the mRNA level of HSP47 was also found elevated in the PBMC from SSc, suggesting HSP47 might become a serum marker of the fibrosis activity of SSc. Since HSP47 has intracellular location and does not secrete out of cells, it seems that the elevated sera level of HSP47 in mixed connective tissue disease (MCTD) and rheumatic diseases (Yokota et al., 2003) and the higher plasma level of HSP47 detected in SSc in our study are produced by the apoptotic cells under the inflammatory and fibrotic condition. Whereas our further analysis demonstrated that both mRNA and plasma levels of HSP47 had a significant correlation with the presence of anti-centromere antibody, which is usually more common in patients with limited scleroderma, and a biomarker for better prognosis for SSc patients (Mehra et al., 2013). Thus, an increased level of HSP47 might be a potential serum biomarker for better prognosis of SSc patients.

Further analysis found the transcription levels of both HSP47 and collagen genes were significantly elevated in the PBMCs of SSc patients. To the best of our knowledge, this is the first report that the expressions of HSP47 and collagen genes are up-regulated in the PBMC of SSc patients, which may provide an explanation for the systemic fibrosis of SSc due to the circulating of PBMC in the body. Previous studies have described a newly characterized collagen-secreting cell type named circulating fibrocyte in the PBMC, which was associated with various fibrotic disorders, such as scleroderma and pulmonary diseases (Quan et al., 2004; Quan et al., 2006). Thus, we hypothesized that the circulating fibrocytes from PBMCs might produce the collagen and HSP47 in SSc patients and contributed to fibrogenesis of SSc.

Myofibroblasts, characterized by expressing α-SMA (Jercan et al., 2012) is the major cell type responsible for secretion of the ECM proteins (e.g., collagen isoforms, cellular fibronectin, etc.) (Kach et al., 2013), and a significant increase in myofibroblasts has been previously observed in the skin of scleroderma patients (Krieg et al., 2007). HSP47 was found mainly expressed in the α-SMA-positive cells (Fig. 2), i.e. myofibroblasts (Abraham et al., 2009) and the number of HSP47-positive cells determined by IHC in the present study was also increased in the skin tissues from SSc patients, suggesting a potential role of HSP47 in the development of SSc fibrosis. Up-regulation of HSP47 was further confirmed in the dermal fibroblasts from SSc patients, and was in accordance with the high expression levels of type I and type III procollagens in the present study (Fig. 2). The up-regulation of HSP47 was also observed in the BLM-induced SSc mouse model, and was correlated with the increased production of collagen, further confirming its role in the excessive production and deposition of collagen in SSc.

TGF-β increases the production of ECM and induces fibrosis (Xiao et al., 2012; Yan et al., 2009) and its expression is highly related to the pathogenesis of scleroderma. TGF-β can also induce HSP47 expression by enhancing the heat shock element binding activity of heat shock transcription factor (Sasaki et al., 2002). Our results showed that HSP47 was up-regulated by TGF-β in a dose-dependent manner; meanwhile, collagen genes were also up-regulated in the same manner (Fig. 5), suggesting that TGF-β signaling might be involved in the modulation of HSP47 expression during SSc fibrosis development. Although TGF-β plays a fundamental role in the progression of fibrosis, it is also a cytokine with pleiotropic functions involving in many physiological processes, and not suitable as a target for fibrosis therapy. Little progress has been made in developing TGF-β pathway inhibitors as anti-fibrotic drugs. Therefore, therapeutic strategies for SSc and some other fibrotic disorders may be designed by blockade of HSP47 expression, the downstream effector of TGF-β signaling, to ameliorate fibrosis. As a collagen-specific chaperone, HSP47 is implicated in the posttranslational stage of collagen, which is the mature and/or secretion of pro-collagen molecules. We found that the expression of HSP47 did have an effect on the transcription level of collagens. It was plausible that blockade of HSP47 expression could result in the accumulation of insoluble aggregates within the ER of the cells (Ishida et al., 2006), which provided a negative feedback of transcriptional regulation of collagens. Thus, HSP47 inhibition with siRNA or some other inhibitors might be a good choice for the treatment of fibrotic disorders without disrupting the cellular homeostasis and with fewer side effects.

In the same time, we have noticed that knockdown of *Hsp47* significantly prevented induction of the lower band detected by anti-ColI antibody, but slightly prevented that of the upper band, which may due to the following reasons. The upper band is α1(I) and the lower band is α2(I). It was reported that TGF-β interacts with Smad binding element (SBE) in the promoter origin of the collagen genes (Derk and Jimenez, 2003; Leask and Abraham, 2004; Verrecchia et al., 2006), making no difference in the expressions of α2(I) and α1(I) genes by exogenous TGF-β stimulation. However, treatment with antisense oligonucleotide against Hsp47 efficiently blocked the production of procollagen α2(I) protein other than α1(I) protein (Ohba et al., 2003). Thus, the knockdown of *Hsp47* might only affect the expression of α2(I), which is the lower band in the WB figure.

In summary, the results provided herein present clear evidence that HSP47 is involved in fibrogenic process of scleroderma and collagen expression is regulated, at least partially, by HSP47. The expression of HSP47 is confirmed to be regulated by the profibrogenic cytokine TGF-β, and further investigations should be performed to validate this potential prognosis biomarker for SSc patients and important therapeutic target for fibrosis disorders.

## MATERIALS AND METHODS

### Patients

Fifty-four Chinese patients who were fulfilled the criteria for diagnosis with SSc were enrolled from Shanghai Traditional Chinese Medicine-Integrated Hospital (Table 1) Masi (1980). Laboratory tests were conducted for all patients to confirm the diagnosis for SSc. Thirty-eight age- and gender-matched healthy individuals were enrolled as normal controls. All the participants provided written informed consent for the sample collection and subsequent analysis. The present study was approved by and carried out in accordance with the guidelines of the School of Life Sciences, Fudan University.

**Table 1:** Clinical and laboratory data of patients with SSc

Age (years), mean ± SD	46 ± 11.4
Gender	
Female	39
Male	15
Disease subset	
Limited SSc	38
Diffuse SSc	16
Laboratory findings	
Anti-topoisomerase	30 (57.7%)
Anti-centromere antibody	10 (20.0%)
Anti-U1RNP antibody	10 (19.6%)
Anti-RNA polymerase antibody	1 (2.0%)
Lung involvement	38 (80.8%)

### Establishment of skin fibrosis mouse model

Specific pathogen-free, female C3H mice (7 weeks old; Sino-British Sippr/BK Lab Animal Ltd., Shanghai, China) were provided *ad libitum* water and pelleted food at the Animal Centre of State Key Laboratory of Genetic Engineering, School of Life Sciences, Fudan University. Bleomycin (BLM; Nippon Kayaku, Tokyo, Japan) was dissolved in saline at 200 μg/mL. Filter-sterilized BLM or saline was injected (100 μL) subcutaneously into the same site of the shaved upper back daily for 3 weeks. All the animal protocols were approved by the School of Life Sciences, Fudan University, China (Wu et al., 2014).

### Primary human skin fibroblasts culture

Skin biopsy specimens were obtained from five SSc patients (four females and one male; three diffuse cutaneous SSc (dcSSc) and two limited cutaneous (lcSSc), the average age was 21.6 ± 6.6 years (mean ± SD), who were fulfilled the criteria for SSc as defined by the American College of Rheumatology (formerly the American Rheumatism Association) (1980). Skin biopsy specimens from normal controls with no history of autoimmune and other dermal diseases were used as control comparators. Skin samples were transported in Dulbecco’s modified Eagle’s medium (DMEM) supplemented with 10% fetal calf serum (FCS) for processing the same day. The skin samples were washed in 75% ethanol, phosphate buffered saline (PBS), and DMEM with 10% FCS. Cultured fibroblast strains were established by mincing tissues and placing them into 60-mm culture dishes secured by glass overlays. The third to fifth passage of human dermal fibroblasts were placed into 12-well culture plates at the density of 1 × 10^5^ cells per well for gene and protein expression assays.

### Cell culture and exposure to TGF-β

Mouse embryonic NIH/3T3 fibroblasts were cultured in DMEM medium supplemented with 10% FCS at 37°C in a 5% CO_2_ humidified incubator. After 12 h of serum starvation in serum-free media, fibroblasts were exposed to recombinant human TGF-β (R&D Systems, Inc., Minneapolis, MN, USA) at gradient concentrations of 0, 1.25, 2.5, 5.0, and 10.0 ng/mL for 12, 24, 36, and 48 h for RNA and protein collection.

### Preparation and transfection of *Hsp47* siRNA

Mouse *Hsp47* siRNA and non-silencing siRNA were purchased from Dharmacon (USA). For siRNA transfection, NIH/3T3 fibroblasts in a 12-well culture plate were cultured with or without TGF-β for 24 h, and then transfected with *Hsp47* siRNA or non-silencing siRNA using Lipofectamine RNAiMAX (Invitrogen) at a concentration of 50 nmol/L. The cells were harvested 48 h after transfection for gene and protein expression assays.

### Histologic analysis

For the assessment of histopathological changes, skin tissues were fixed with 4% paraformaldehyde and embedded in paraffin. Then 4-μm-thick skin sections were stained with hematoxylin/eosin or Masson’s trichrome staining for better visualization of the tissue structure. Dermal thickness was analyzed with a Nikon Eclipse 80i microscope (Nikon, Badhoevedorp, The Netherlands) by measuring the maximal distance between the epidermal-dermal junction and the dermal-subcutaneous fat junction at 4 different skin sections in each mouse, as previously described (Yoshizaki et al., 2010). The evaluation was performed by two independent examiners.

### RT-PCR and quantitative RT-PCR analysis

Total RNA was extracted from the skin of mice, human skin fibroblasts (described in the **Primary human skin fibroblasts culture** section above), and peripheral blood mononuclear cell isolated from patients and normal individuals’ blood (described in the **Patients** section above) using Trizol (Invitrogen, Carlsbad, CA, USA). One microgram of total RNA was subjected to cDNA synthesis using the High Capacity cDNA Reverse Transcription Kit (Applied Biosystems) according to the instructions of the manufacturer. The specific primers for each gene were designed using Primer 5 and synthesized by Generay Biotech Co., Ltd. (Shanghai, China). The RT-PCR amplification was conducted using a SYBR Green I PCR Kit (TaKaRa, Shiga, Japan) according to manufacturer’s instructions. The reaction was carried on ABI Prism 7900 Detector System (Applied Biosystems). RT-PCR conditions were 95°C for 3 min, followed by 40 cycles of 95°C for 15 s, 60°C for 40 s, and the conditions for getting the dissociation curve was 95°C for 15 s, 60°C for 15 s, 95°C for 15 s. The data obtained from the assays were analyzed with SDS 2.3 software (Applied Biosystems). For each sample, the relative gene expression was calculated using a relative ratio to *Gapdh*/*GAPDH*.

### Collagen measurements

Total soluble collagen from mice skins and cell culture supernatants was quantified using the Sircol collagen assay (Biocolor, Belfast, UK). In detail, collagen of the mice skins was extracted overnight with 2 mg/mL pepsin in 5 mol/L acetic acid, while collagen secreted from cultured cells were collected from the supernatant of confluent cells which were incubated for 24 h with 1 mL DMEM and 5% FCS in 25-cm^2^ culture dishes. Then, 1 mL of Sirius red dye, an anionic dye that reacted specifically with basic side chain groups of collagens under assay conditions, was added to 20 μL collagen solution of mouse skin or 400 μL of cell supernatant, and incubated with gentle rotation for 30 min at room temperature. After centrifugation at 12,000 ×*g* for 10 min, the collagen-bound dye was re-dissolved with 1 mL of 0.5 mol/L NaOH, and the absorbance at 555 nm was measured. The absorbance is directly proportional to the amount of collagen in skin tissues and cell supernatants.

### ELISA for HSP47 in plasma

The concentrations of HSP47 in the plasma of SSc patients were measured using an ELISA kit (Abcam, Hong Kong, Ltd.) according to the manufacturer’s instructions.

### Western blot analysis

The cell lysates extracted from the skin tissues of mice and the cultured cells were used for immunoblotting analysis. Total protein concentration was measured using the BCA protein kit (Vazyme, China) with bovine serum albumin (BSA, Sigma-Aldrich) as the standard protein. Equal amounts of protein from each sample were subjected to 10% SDS PAGE gels electrophoresis, and subsequently transferred to PVDF membranes (Millipore). The membranes were blocked with 5% milk at room temperature for 1 h. Then the membranes were incubated with rabbit anti-HSP47 monoclonal antibody (1:1000) (Abcam, Hong Kong, Ltd.), rabbit anti-alpha-SMA monoclonal antibody (1:50000) (Millipore, clone E184), goat anti-Collagen type I polyclonal antibody  (1:500) (Millipore), rabbit anti-mouse anti-Collagen type I polyclonal antibody (1:500; Millipore) or internal control GAPDH antibody (Cell Signaling Technology) at 4°C overnight. After three washes with TBST for 30 min, the PVDF membranes were incubated with the horseradish peroxidase-conjugated secondary antibody of goat anti-rabbit, rabbit anti-goat or goat anti-mouse lgG for 1 h at room temperature. The protein bands were visualized using an enhanced chemiluminescence system (Thermo) and the intensity of bands was quantified using ImageQuantTL software (General Electric Company).

### Immunohistochemical staining of α-SMA and HSP47

For immunohistochemistry assay, the primary antibodies used were anti-α-SMA (1:500; Millipore, clone E184) and monoclonal anti-HSP47 antibodies (1:200; Abcam, Hong Kong, Ltd.). Skin tissues obtained from five patients (five females; median age 27 years, range 13–43 years) and normal controls were formalin-fixed and paraffin-embedded. Skin sections were deparaffinized and incubated with 5% bovine serum albumin for 60 min. Cells positive for α-SMA and HSP47 were detected by incubation with the primary antibody for 2 h at room temperature followed by incubation with 3% hydrogen peroxide for 10 min. Goat anti-rabbit lgG labeled with horseradish peroxidase were used as secondary antibodies. The expression of α-SMA and HSP47 was visualized with 3,3’-diaminobenzidinetetrahydrochloride (DAB-4HCl).

### Statistical analysis

An independent two group *t*-test or one-way Analysis of Variance (ANOVA) with LSD multiple comparison tests were used to evaluate significant differences between groups. *P* values less than 0.05 were considered significant.

## Abbreviations

α-SMA: α-smooth muscle actin
BLM: bleomycin
COLI: type I collagen
dcSSc: diffused cutaneous SSc
ECM: extracellular matrix
HE: hematoxylin and eosin
HSP47: heat-shock protein 47
lcSSc: limited cutaneous SSc
MMP: extracellular matrix metalloproteinase
PBMC: peripheral blood mononuclear cells
Sa: saline
siRNA: small interfering RNA
TGF-β: transforming growth factor factor beta
TIMP: tissue inhibitor of metalloproteinase
